# miR-15b-5p Promotes Growth and Metastasis in Breast Cancer by Targeting HPSE2

**DOI:** 10.3389/fonc.2020.00108

**Published:** 2020-02-26

**Authors:** Balu Wu, Guohong Liu, Yanxia Jin, Tian Yang, Dongdong Zhang, Lu Ding, Fuling Zhou, Yunbao Pan, Yongchang Wei

**Affiliations:** ^1^Department of Hematology, Zhongnan Hospital of Wuhan University, Wuhan University, Wuhan, China; ^2^Department of Radiology, Zhongnan Hospital of Wuhan University, Wuhan University, Wuhan, China; ^3^Department of Clinical Oncology, Zhongnan Hospital of Wuhan University, Wuhan University, Wuhan, China; ^4^Department of Laboratory Medicine, Zhongnan Hospital of Wuhan University, Wuhan University, Wuhan, China; ^5^Hubei Key Laboratory of Tumor Biological Behaviors, Department of Radiation and Medical Oncology, Zhongnan Hospital of Wuhan University, Wuhan, China

**Keywords:** breast cancer, microRNA, miR-15b-5p, HPSE2, biomarker

## Abstract

MicroRNAs (miRNAs) can participate in many behaviors of various tumors. Prior studies have reported that miR-15b-5p in different tumors can either promote or inhibit tumor progression. In breast cancer, the role of miR-15b-5p is unclear. The main objective of this paper is to explore miR-15b-5p effects and their mechanisms in breast cancer using both *in vitro* and *in vivo* experiments. This study showed that miR-15b-5p expression was upregulated in breast cancer compared with normal breast tissue and was positively correlated with poor overall survival in patients. Knockdown of miR-15b-5p in MCF-7 and MD-MBA-231 breast cancer cells restrained cell growth and invasiveness and induced apoptosis, whereas overexpression of miR-15b-5p achieved the opposite effects. We next revealed a negative correlation between miR-15b-5p and heparanase-2 (HPSE2) expression in breast cancer. Knockdown of miR-15b-5p significantly increased HPSE2 expression at both mRNA and protein levels in breast cancer cells *in vitro*. The underlying mechanisms of miR-15-5p in breast cancer were investigated using luciferase activity reporter assay and rescue experiments. In addition, miR-15b-5p knockdown significantly inhibited tumor growth in a xenograft model in mice. In summary, we showed that miR-15b-5p promotes breast cancer cell proliferation, migration, and invasion by directly targeting HPSE2. Accordingly, miR-15b-5p may serve both as a tool for prognosis and as a target for therapy of breast cancer patients.

## Introduction

Breast cancer is one of the most commonly diagnosed types of cancer in women, and it is the leading cause of cancer mortality among women worldwide (1, 2). Breast cancer cells have strong invasive ability and often form distant metastases (3, 4). Despite the great progress achieved in therapeutics, relapse and metastasis are still the leading reasons for death in breast cancer patients (5). Therefore, finding specific molecules which can serve as markers for early diagnosis and prognosis and can become therapeutic targets has become an important research direction.

miRNAs are small non-coding RNAs which are involved in the initiation and progression of various types of cancer (6). Previous researches show a role for miRNAs in regulation of proliferation, invasion, metastasis, and apoptosis (7, 8). Investigation of the mechanisms underlying the regulation of breast cancer by miRNAs is essential (9). The miRNA miR-15b-5p, a mature miRNA which is spliced from the 5′-end of pre-miR-15b (10), has been reported to be expressed in many cancers, including gastric cancer (11), colorectal cancer (12), prostate cancer (13), bladder cancer (14), and hepatocellular carcinoma (15). In these tumors, miR-15b-5p promotes proliferation, decreases apoptosis, induces tumor metastasis and recurrence, and is associated with poor patient prognosis. The role of miR-15b-5p in BC, however, is rarely reported. It has been reported that miR-15b is highly expressed in breast cancer and promotes its progression by binding to the 3′ untranslated region (UTR) of metastasis suppressor protein 1 (MTSS1) and downregulating its expression (16). Another study has shown that miR-15b is downregulated in an inducible model of cancer stem cell (CSC) formation in a breast cell line and inhibits CSC growth (17). However, the CSC is established by non-transformed breast epithelial cells (MCF-10A) carrying an inducible Src oncogene (ER-Src) with tamoxifen. In fact, it is not really a tumor cell. Thus, the role of miR-15b in breast cancer deserves further study.

The primary goal of this work is to determine the effects of miR-15b-5p in breast cancer tissues and cell lines and develop an understanding of their underlying mechanisms. Our findings showed that miR-15b-5p expression significantly upregulated in breast cancer tissues. We hypothesized that miR-15b-5p functions as an oncogene in breast cancer when overexpressed. We found that overexpression of miR-15b-5p promoted breast cancer cell proliferation, colony formation, invasion, and metastasis and suppressed apoptosis. Knockdown of miR-15b-5p produced the opposite effects. Using microarray analysis and bioinformatics prediction tools, we discovered that its target is heparanase-2 (HPSE2). Additionally, it was shown that miR-15-5p directly targeted the 3′ UTR of HPSE2 and restrained its expression. Then, it was confirmed using rescue experiments that miR-15-5p directly targeted HPSE2. Thus, our results confirm that miR-15b-5p may mediate its carcinogenic effects in breast cancer by inhibiting HPSE2 expression. Altogether, our results revealed that miR-15b-5p accelerates breast cancer progression via disinhibiting HPSE2, simultaneously providing a novel diagnostic and prognostic marker and a potential therapeutic target.

## Materials and Methods

### Microarray Analysis and Bioinformatic Analysis

In total, six samples from three breast cancer patients comprising three invasive ductal carcinomas and three neighboring areas of normal breast tissue were studied by microarray analysis by a professional company (Genminix Information Co., Ltd., Shanghai, China). The differentially expressed miRNAs were identified in breast cancer and normal samples by fold change filtering, and the heatmap was used to plot the expression profiles. We exported the raw microarray data to the Gminix-Cloud Biotechnology Information website GCBI (Genminix Information Co., Ltd., Shanghai, China) for further analysis, including prediction of microRNA target genes and miRNAs–gene network analysis. The intensity of the signal was calculated after background subtraction, and replicated spots on the same slide were averaged to obtain the median intensity. The relationship between miR-15b-5p and breast cancer prognosis was explored by analyzing the miRNA expression profile of breast cancer specimens in The Cancer Genome Atlas (TCGA) database (18). The targets of miR-15b-5p were predicted using TargetScan human version 7.2 (http://www.targetscan.org/vert_72/) (19). This revealed that the 3′ UTR of HPSE2 may be complementary with the seed sequences of miR-15b-5p.

### Chemicals and Reagents

Cell culture medium RPMI-1640 and Dulbecco's Modified Eagle Medium (DMEM) were bought from Invitrogen (Carlsbad, CA, USA), and fetal bovine serum (FBS) was purchased from Gibco (Grand Island, NY, USA). A total RNA extraction kit and transfection reagent Lipofectamine 2000 were bought from Invitrogen (Carlsbad, CA, USA). si-NC, miRNA mimic, miRNA inhibitor, and inhibitor NC were designed and synthesized by GenePharma (Shanghai, China). Plasmid vectors expressing HPSE2 or the negative control (NC) were constructed by GenePharma (Shanghai, China). The anti-HPSE2 antibody was purchased from Abcam Biotechnology (Cambridge, UK), and anti-GAPDH was purchased from Proteintech (Wuhan, China).

### Cell Lines and Cell Cultures

Two human breast cancer cell lines (MDA-MB-231 and MCF-7) and one normal human embryonic kidney cell line (293T) cell were used in this study. All were purchased from the Type Culture Collection of the Chinese Academy of Sciences (Shanghai, China). Cells were cultured in RPMI-1640 or DMEM (Invitrogen; Carlsbad, CA, USA) containing 10% fetal bovine serum (Gibco; Grand Island, NY, USA) and 1% penicillin–streptomycin sulfate (Invitrogen, Carlsbad, CA, USA). All cell lines were incubated with 5% CO_2_ at 37°C.

### Tissue Microarray and IHC and ISH Analyses

Tissue microarrays were obtained from Outdo Biotech Co., Ltd. (Shanghai, China). Immunohistochemical (IHC) studies of HPSE2 and *in situ* hybridization (ISH) analyses of miR-15b-5p were performed on breast cancer samples within the tissue microarray. The paraffin-embedded tissues were sliced at a thickness of 4 μm. After dewaxing and hydration, the tissue sections were incubated with 3% H_2_O_2_ for 30 min to block the endogenous peroxidase activity. Antigen retrieval was accomplished through repeated cooling and heating, and non-specific binding was blocked with 5% bovine serum albumin. Then the sections were incubated with primary antibodies overnight at 4°C. Anti-HPSE2 (ab97807) was purchased from Abcam (Cambridge, UK) and used at a dilution of 1:100. After three 5-min phosphate-buffered saline (PBS) washes, sections were treated with biotinylated secondary antibody (Abcam) for 1 h, following which streptavidin–horseradish peroxidase (HRP) was incubated for 20 min. HPSE2-positive cells were observed with a diaminobenzidine substrate. Then the slides were observed under a microscope (Olympus BX51, Olympus Optical, Tokyo, Japan).

To perform ISH staining on the tissue microarray, we purchased the digoxigenin (DIG)-labeled miR-15b-5p probe from Exiqon. Histologic sections were hybridized with a dual-labeled RNA probe for 2 h. It is then detected with an anti-DIG antibody. miR-15b-5p was considered to be positive when either cytoplasm or nuclei of cancer cells was stained. The positivity of the specimen was taken as the estimated proportion of positively stained cells.

### Cell Transfection and Transduction

Three cell lines (MCF-7, MDA-MB-231, and 293T) were used for cell transfection. Cells (5 × 10^5^) were plated onto six-well-plates 24 h before transfection. Negative control, miR-15b-5p mimic, miR-15b-5p inhibitor, NC inhibitor, and HPSE2 siRNA were transfected, respectively, via Lipofectamine 2000 reagent (Invitrogen, Carlsbad, CA, USA) per the manufacturer's instructions when the cell density reached 50–60%. The final concentration of miRNA inhibitors, miRNA mimics, or NC was 50 nM, and the final concentration of siRNA was 30 nM. After 4 h of transfection, a conventional medium was used to replace the Opti-MEM medium (Gibco, Grand Island, NY, USA) without antibiotics. Cells were harvested for further tests 24–48 h after transfection. For the selection of stable cell lines, lentivirus was transduced into MDA-MB-231 as previously described (20). Lentivirus expressing hsa-miR-15b-5p inhibitor was bought from GenePharma and was used to infect MDA-MB-231 cells, and cells were selected using medium containing 1.0 μg/ml puromycin.

### qRT-PCR

Total RNA from harvested cells was isolated by the TRIzol reagent (Invitrogen; Thermo Fisher Scientific, USA) as previously described (21). The purity and quality of total RNA were measured using a NanoDrop ND-2000 spectrometer (NanoDrop Technologies, Wilmington, DE, USA). Total RNA (500 ng) reverse transcription was performed using a reverse transcription kit (Takara, Dalian, China). qRT-PCR was performed with an Applied Biosystems 7500 systems (Applied Biosystems, Foster City, CA, USA). U6 and GAPDH served as endogenous controls. The expression of miR-15b-5p and HPSE2 were evaluated using the 2^−ΔΔCq^ method. The primer sequences used in this study are listed in Table S2.

### Western Blot Assay

Forty-eight hours after cell transfection, the cells were washed with PBS and then lysed in radioimmunoprecipitation assay (RIPA) lysis buffer (Beyotime Institute of Biotechnology, Nantong, China) containing 1 mmol/L of phenylmethanesulfonyl fluoride (Multi Sciences, Wuhan, China) on ice for 30 min and then centrifuged at 12,000 × g at 4°C for 20 min to extract the protein. The quality of the total protein was determined via the bicinchoninic acid (BCA) method. Briefly, 30 μg of proteins measured by a BCA protein quantitation kit (Thermos, Waltham, MA, USA) was separated using 10% SDS-PAGE. Subsequently, the gels were transferred to 0.22-μm polyvinylidene fluoride or polyvinylidene difluoride (PVDF) membranes (Millipore Corp., MD, USA), and the membranes were blocked with 5% skim milk for 1 h at room temperature. The blot was then probed with rabbit polyclonal antibodies against HPSE2 (1:1,000, Abcam, Cambridge, UK) or GAPDH (internal positive control for immunoblots at 1:5,000, Proteintech, Wuhan, China) and incubated at 4°C overnight. Then the membranes were washed three times, incubated with HRP-conjugated goat anti-rabbit or goat anti-mouse IgG secondary antibodies as appropriate (1:5,000, Proteintech, Wuhan, China) for 2 h at room temperature. Specific protein bands were detected via an enhanced chemiluminescence (ECL) kit (BosterBio, Wuhan, China) on a Western Chemiluminescent Imaging System (Tanon 5200, Wuhan, China).

### Cell Proliferation Assay

Cell proliferation ability was measured via the 3-(4,5-dimethylthiazol-2-yl)-2,5-diphenyltetrazolium bromide (MTT) assay. Briefly, 24 h after transfection, the cells were seeded into 96-well-plates at 1 × 10^3^ cells per well. Then 20 μl of 0.5 mg/ml MTT solution was added to each well once daily for 6 successive days. The cells were incubated at 37°C for 4 h, and then 150 μl of dimethyl sulfoxide was added to each well. Absorbance values at 570 nm were read using a multifunctional enzyme-linked immunosorbent assay microplate reader (SpectraMax, M2, CA, USA). The experiments were repeated at least three times.

### Colony Formation Assay

The clonogenicity of a single cell was displayed via a colony formation assay. In brief, the transfected cells were seeded into six-well plates at a concentration of 200 cells per well (MCF-7) or 500 cells per well (MDA-MB-231). The culture medium was changed twice per week. They were cultured for 10–14 days. After that, cells were washed with PBS at least twice and subsequently fixed and stained with 0.1% crystal violet for 15 min. The colonies (consisting of ≥50 cells each) were scored by counting with an inverted microscope. Cell viability was calculated as (number of colonies in the treatment group/the number of colonies in the control group) × 100%.

### Wound Healing Assay and Transwell Assay

Wound-healing assay was used to evaluate the migration ability of cancer cells. In brief, the transfected cells were cultured in six-well plates (5 × 10^5^ cells per well). When the cell density was close to 90%, the confluent cell monolayer was scratched with a sterile 200-μl pipette tip in a straight line, washed with PBS, and further cultured in medium containing 1% FBS for 24 h. Closure of each scratch was photographed under a light microscope (Olympus IX50, Tokyo, Japan) at 0, 12, and 24 h. Image-Pro Plus software (Version 5.1, Media Cybernetics, Inc., USA) was used to calculate the ability of cellular migration in each group.

Cell invasion assay was implemented using BD Control Inserts and BioCoat Matrigel Invasion Chambers (BD Biosciences, NYC, USA). Cells were seeded in the upper chamber at 5 × 10^3^ cells per well in 100 μl of serum-free media, and the lower chamber was filled with 600 μl of complete medium. After 24 h of incubation, the cells in the upper chamber were wiped with a cotton swab, and the invaded cells were fixed with methanol for 15 min, stained with 0.1% crystal violet, and scored by counting with an inverted-contrast microscope (Olympus IX50, Tokyo, Japan) using at least five random fields of view. The experiments were repeated at least three times.

### Flow Cytometry Analysis of Cell Apoptosis

Cell apoptosis was detected using an annexin V–fluorescein isothiocyanate (FITC)/propidium iodide (PI) apoptosis detection kit (Kaiji, Nanjing, China). Briefly, the treated cells were harvested by high-speed centrifugation (1,500–2,000 rpm) and washed twice with ice-cold PBS. Then cells were resuspended in 500 μl of binding buffer. Then cells were stained with 5 μl of propidium iodide and 5 μl annexin V–FITC at room temperature for 15 min in the dark. Flow cytometry (Becton Dickinson, CA, USA) was used to detect cell apoptosis. Finally, the results were analyzed using Flow Jo software (Flow Jo, Ashland, OR, USA).

### Dual-Luciferase Assay

The wild-type (WT) and mutant (Mut) binding sequences of miR-15b-5p on the 3′ UTR of HPSE2 were synthesized (GenePharma, Shanghai, China) and cloned into the pMIR-reporter vector (Invitrogen). Cells (MCF-7 and 293T) were seeded into 96-well-plates at a density of 1 × 10^4^ cells per well. When the cells spread to nearly 80–90% of the bottom of 96-well-plates, they were transfected with luciferase reporters, either WT or Mut HPSE2 3′ UTR, with or without miR-15b-5p mimics or NC by Lipofectamine 2000 (Invitrogen, Carlsbad, CA, USA). Forty-eight hours later, the quantitative analysis of firefly and renilla luciferase activity via the Dual-Glo Luciferase Reporter Assay system (Promega, Madison, WI, USA) on a multifunctional enzyme-linked immunosorbent assay microplate reader (Turner BioSystems, Sunnyvale, CA, USA) per the manufacturer's operation manual. For data analysis, firefly luciferase activity was normalized to the corresponding renilla luciferase activity. All experiments were implemented in triplicate and repeated three times.

### Mouse Model

For the orthotopic xenograft tumor model, 5-week-old female BALB/C nude mice were purchased from Beijing HFK Bioscience (Beijing, China) and raised in a specific pathogen-free, climate-controlled facility at the Animal Experiment Center of Wuhan University (Wuhan, China). The mouse flank was subcutaneously injected with 200 μl of 5 × 10^6^ MDA-MB-231 cells stably down-expressing either miR-15b-5p or miR-control. Mice were checked every 3 days for xenograft development. Tumor volume was calculated twice per week as length × width^2^/2. Five weeks later, nude mice were euthanized, and tumor volumes were again measured. All procedures for animal experiments were approved by the Animal Care and Use Committee of Wuhan University (ethical approval number is AUP2017070).

### Data Mining

The data of the expression of miR-15b-5p and HPSE2 in breast cancer were acquired from ENCORI (starbase.sysu.edu.cn), a platform that is designed for decoding Pan-Cancer Networks of lncRNAs, miRNAs, pseudogenes, snoRNAs, RNA-binding proteins (RBPs), and all protein-coding genes by analyzing their expression profiles across 32 cancer types (~10,000 RNA-seq and ~9,900 miRNA-seq samples) integrated from TCGA project. A total of 1,109 cases of breast cancer patients were included in our study. Besides, 113 cases of non-tumor breast tissues were also extracted from the TCGA dataset. The relevance of overall survival (OS) and relapse-free survival (RFS) rates with the expression of miR-15b and HPSE2 were analyzed by the online analysis tool Kaplan–Meier Plotter (https://kmplot.com/analysis/). The system includes gene chip and RNA-seq data—sources for the databases include GEO, EGA, and TCGA. Its primary purpose is a meta-analysis-based discovery and validation of survival biomarkers.

### Statistical Analysis

All of the data presented are expressed as the mean ± standard deviation (SD). The indicators of paired samples were analyzed with a paired-sample *t*-test, while those of unpaired samples were analyzed by one-way ANOVA for three or more groups or by the *t*-test for two groups. To examine the prognostic significance of miR-15b-5p and HPSE2 in breast cancer patients, Kaplan–Meier survival analysis (*P*-value of the log-rank test) was used to explore the association between the OS/RFS rate and miR-15b-5p/HPSE2 levels in tumors of breast cancer patients. Long and short OS and disease-free survival (DFS) were discriminated based on the best cutoff value recommended by the website. Pearson's correlation analysis was applied to evaluate the relationship between miR-15b-5p and HPSE2 mRNA expression in breast cancer tissues. SPSS (version 22.0; IBM Corp., Armonk, NY, USA) and GraphPad (version 7.0; San Diego, CA, USA) were used for statistical analysis. *P* < 0.05 was considered to be statistically significant.

## Results

### Microarray Expression Profiles and Correlation of miR-15b-5p/HPSE2 Expression With Clinical Outcome in Breast Cancer

**Figure 1:**
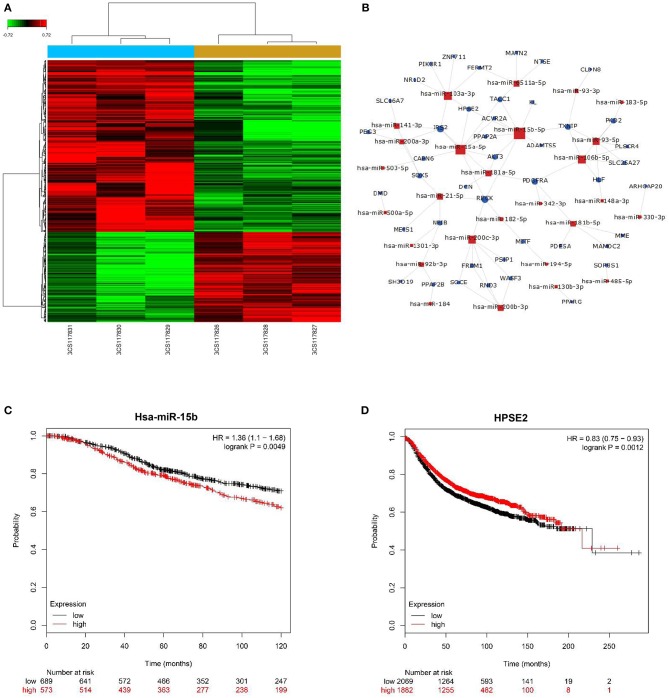
miRNA expression profiling in three paired breast cancer and adjacent normal tissues. **(A)** The heatmap reveals clusters of differentially expressed miRNAs; green indicates relatively low expression, and red indicates relatively high expression. **(B)** miRNAs–gene network was constructed to illustrate the key regulatory functions of the identified miRNAs and their target genes. The size of the circle or square node represents the degree value. A higher degree of gene/miRNAs indicates that it plays a more important role in the signaling network. **(C,D)** Correlation of miR-15b-5p or HPSE2 with overall survival (OS) or relapse-free survival (RFS) of breast cancer patients. The data illustrated are from the Kaplan–Meier Plotter database (http://kmplot.com/analysis/).

We applied microarray analysis to discern the differential expression of miRNAs. A total of 152 significantly differentially regulated miRNAs (fold change ≥1.5, *P* < 0.001) were identified in the profiles, including upregulated and downregulated differential expression miRNAs on breast cancer and adjacent normal tissues (Figure 1A). Figure 1B summarizes the key regulatory functions of the identified miRNAs and their target genes. The network diagram shows an important regulatory relationship between HPSE2 and miR-15b-5p, so we chose it for further study. The online analysis tool Kaplan–Meier Plotter database was used to analyze the relationship between miR-15b-5p or HPSE2 expression level and the OS or RFS of breast cancer. The results indicated that the mean survival time of patients with high miR-15b-5p expression (71 months) was dramatically shorter than that of patients with low miR-15b-5p expression (91 months) (Figure 1C). On the contrary, the mean RFS with high HPSE2 expression (57 months) was markedly longer than the patients with low expression (35 months) in breast cancer (Figure 1D).

### miR-15b-5p and HPSE2 Expression in Breast Cancer

**Figure 2:**
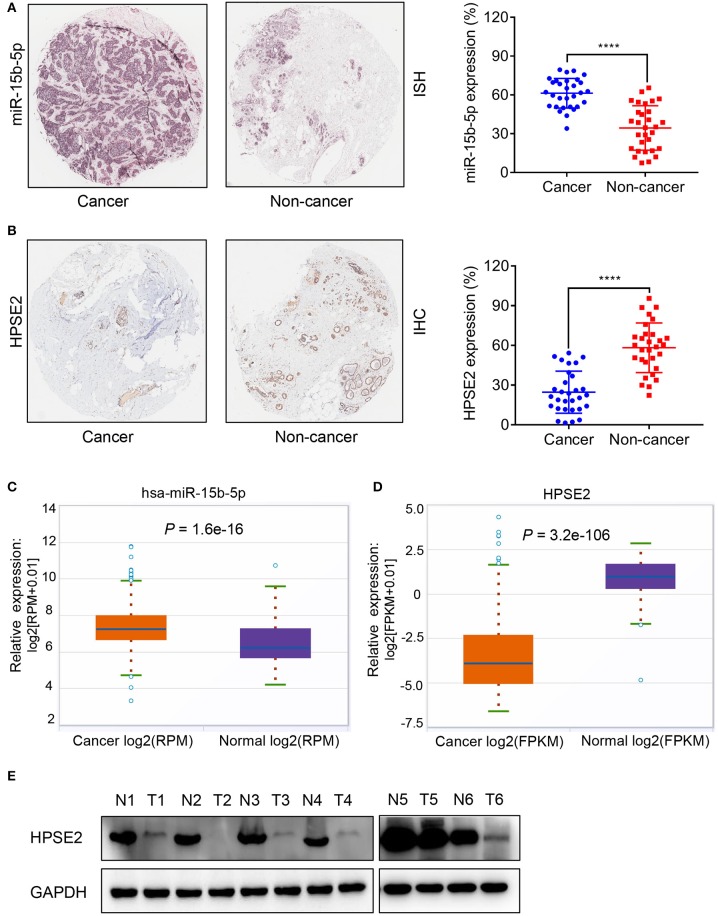
Expression correlation analysis of miR-15b-5p and HPSE2 in breast cancer tissues and matched adjacent non-cancerous tissues. **(A)** *In situ* hybridization (ISH) demonstrating that miR-15b-5p in breast cancer tissues was higher than that in normal tissues (*n* = 30, *P* < 0.0001). **(B)** HPSE2 immunoreactivity in breast cancer tissues was lower than that in normal tissues. The percentage of HPSE2 expression in breast cancer or normal tissues is shown in the figure (*n* = 30, *P* < 0.0001). The patient population represented was from Outdo Biotech Co., Ltd. (Shanghai, China). **(C,D)** miR-15b-5p and HPSE2 expression in normal breast and breast cancer according to the TCGA data. **(E)** HPSE2 proteins have lower expression in breast cancer tissues. The HPSE2 levels in six human breast cancer and paired adjacent normal tissues were measured by western blot analysis. N, paired adjacent normal tissues; T, tumor tissues. Patient information is placed in the Supplementary Materials. (****P < 0.0001).

We used ISH and IHC to evaluate the differential expression of miR-15b-5p and HPSE2 mRNA and protein in breast cancer tissues and adjacent tissues. ISH results indicated that miR-15b-5p was expressed in 61% of breast cancer samples, considerably higher than that of the paired adjacent non-cancerous breast tissue specimens (33%, *P* < 0.0001; Figure 2A). Meanwhile, IHC showed that HPSE2 expression in breast cancer tissues (25%) was prominently lower compared with that in the adjacent normal tissues (58%, *P* < 0.0001; Figure 2B). Similar results were obtained from TCGA databases (Figures 2C,D). Finally, we used six pairs of tumor tissue and adjacent tissues to detect the expression level of HPSE2 protein. This showed that the level of HPSE2 protein in tumor tissues was decreased below that in the adjacent tissues (Figure 2E). These results indicated that miR-15b-5p is overexpressed in breast cancer, while HPSE2 is underexpressed in breast cancer.

### miR-15b-5p Promotes Breast Cancer Cell Proliferation

**Figure 3:**
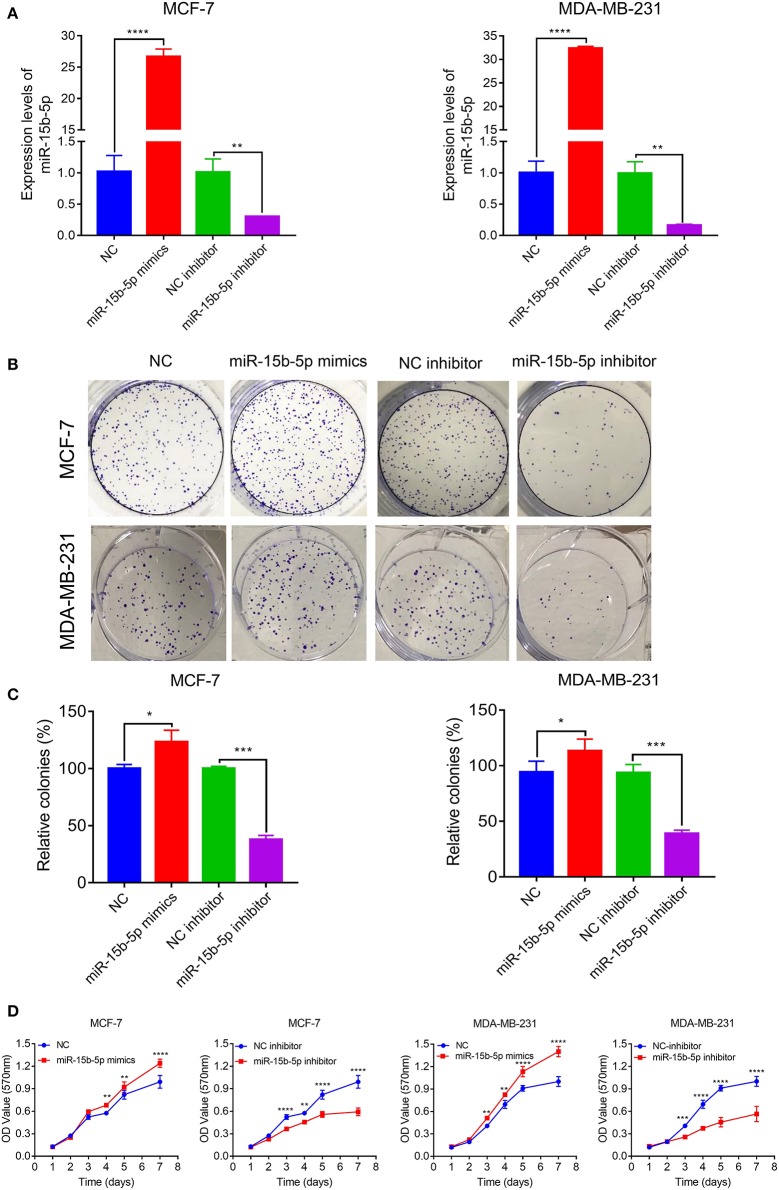
miR-15b-5p promotes breast cancer (BC) cell proliferation. **(A)** Detection of transfection efficiency by qPCR. U6 served as an internal control. **(B,C)** Colony formation assays demonstrated that miR-15b-5p promoted clone formation in BC cell lines following transfection with the miR-15b-5p mimic when compared with negative control (NC). miR-15b-5p inhibited colony formation in BC cell lines following transfection with the miR-15b-5p inhibitor when compared with the inhibitor NC. **(D)** 3-(4,5-Dimethylthiazol-2-yl)-2,5-diphenyltetrazolium bromide (MTT) assay revealed that miR-15b-5p promoted the proliferation of BC cell lines following transfection with the miR-15b-5p mimic when compared with NC, while miR-15b-5p significantly decreased the proliferation of BC cell lines following transfection with the miR-15b-5p inhibitor as compared with the inhibitor NC (*P < 0.05, **P < 0.01, ***P < 0.001, and ****P < 0.0001).

We next explored the function of miR-15b-5p in two breast cancer cell lines. Briefly, cells were transfected with NC, miR-15b-5p mimic, inhibitor control, and miR-15b-5p inhibitor, in that order. Transfection efficiency of miRNA was detected by qPCR (Figure 3A). Then we analyzed the impact of miR-15b-5p through colony formation and MTT assays. The colony formation assay showed that the cell clonality of breast cancer cells was significantly inhibited after knockdown of the miR-15b-5p compared with the control group, while the transfection of miR-15b-5p mimics promoted the colony formation viability of MCF-7 and MDA-MB-231 (Figures 3B,C). Results from MTT assays to assess its effect on the proliferation ability in breast cancer cell lines were consistent with the colony assays (Figure 3D). These results showed that miR-15b-5p could promote the proliferation of breast cancer cells, acting as an oncogene.

### miR-15b-5p Knockdown Induced Breast Cancer Cell Apoptosis

**Figure 4:**
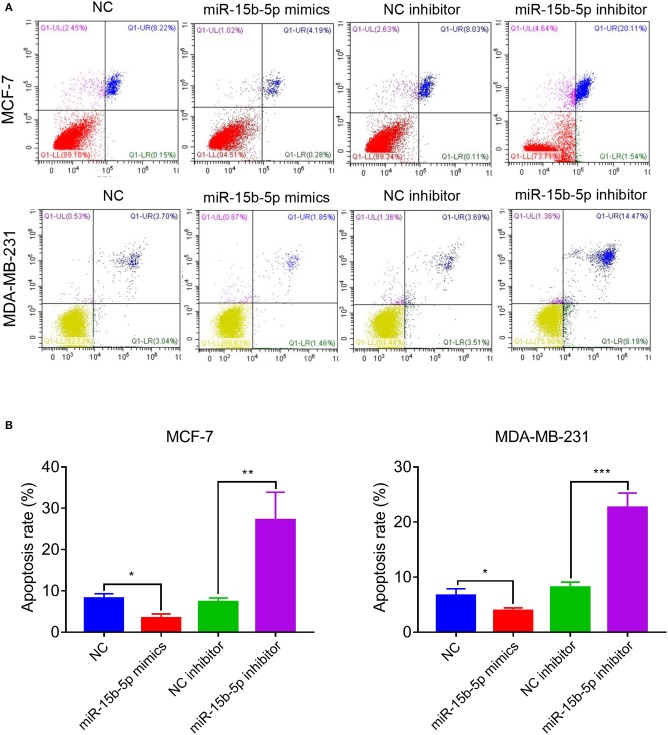
miR-15b-5p knockdown induced cell apoptosis of breast cancer cell lines. **(A,B)** Flow cytometry showed that the apoptotic rates of breast cancer cells were significantly increased following the miR-15b-5p inhibitor when compared with the inhibitor NC (*P < 0.05, **P < 0.01 and ***P < 0.001).

We analyzed the apoptotic rate of cells via flow cytometry. As anticipated, the proportion of apoptotic cells was reduced after transfection of miR-15-5p mimics compared with the NC group, while the opposite result was observed after transfection of miR-15b-5p inhibitor, where the apoptotic rate of tumor cells was significantly increased (Figures 4A,B). In summary, these results suggested that knockdown of miR-15b-5p suppressed apoptosis of breast cancer cells.

### Downregulation of miR-15b-5p Suppresses the Motility and Invasiveness in Breast Cancer Cells

**Figure 5:**
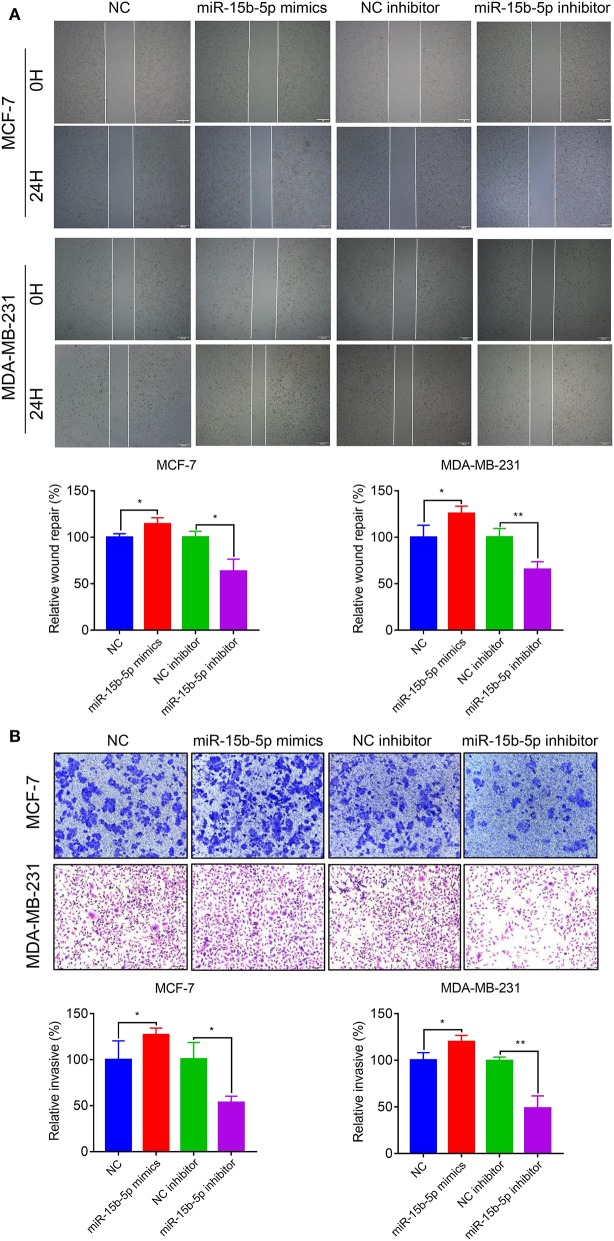
Downregulation of miR-15b-5p suppresses the motility and invasiveness in BC cells. **(A)** Cell migration was measured by scratch-wound assay. Representative images are shown (20×). The results of three independent experiments are summarized. **(B)** Cell invasion was determined by Transwell assay. Representative images are shown (100×). The results of three independent experiments are shown (*P < 0.05 and **P < 0.01).

To explore the effects of miR-15b-5p on breast cancer cell migration and invasion, we performed wound healing and Transwell assays. The wound-healing assay showed that knockdown of miR-15b-5p significantly decreased cell migration distance in MCF-7 and MDA-MB-231 cells (Figure 5A). Transwell assays indicated that miR-15b-5p positively regulates cell invasion. As shown in Figure 5B, inhibiting miR-15b-5p significantly suppressed cell invasion in MCF-7 and MDA-MB-23 cells. These results suggested that miR-15b-5p might act as an oncogene facilitating breast cancer cell migration and invasion.

### HPSE2 Is a Direct Target of miR-15b-5p

**Figure 6:**
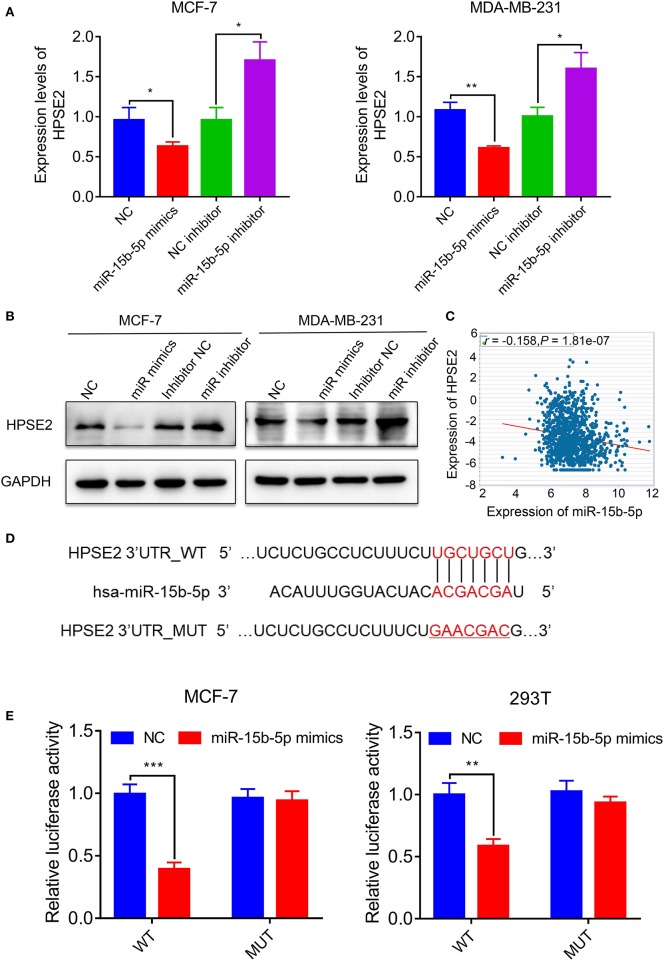
HPSE2 is a target of miR-15b-5p. **(A)** The mRNA level of HPSE2 in breast cancer cells overexpressing or underexpressing miR-15b-5p. **(B)** The protein level of HPSE2 in breast cancer cells overexpressing or underexpressing miR-15b-5p. **(C)** miR-15b-5p expression is correlated with HPSE2 expression in BC from TCGA data. The *r* and *P* values are from Pearson correlation. **(D)** The predicted relationship between miR-15b-5p and HPSE2 via the bioinformatics prediction website. **(E)** The dual luciferase reporter assay demonstrated a targeted relationship between miR-15b-5p and HPSE2 in different cell lines (*P < 0.05, **P < 0.01, and ***P < 0.001).

In order to identify the potential mRNA target of miR-15b-5p, the online analysis tools TargetScan (http://www.targetscan.org/vert_72/) and miRDB (http://www.mirdb.org/) were adopted, and a series of latent targets were selected. Among these candidate targets, HPSE2 immediately drew our attention since it was predicted by miRNAs–gene network analysis. According to the result of qPCR and western blot, knocking down miR-15b-5p significantly enhanced HPSE2 expression at mRNA and protein levels, while upregulating miR-15b-5p inhibited the expression of HPSE2 at mRNA and protein levels (Figures 6A,B). According to TCGA databases, the expression of miR-15b-5p is negatively related to HPSE2 in breast cancer patients (*R* = −0.1580, *P* = 1.81e−07; Figure 6C).

To examine the target relationship, we constructed two reporter plasmids, pmirGLO-HPSE2-wt and pmirGLO-HPSE2-mut. These vectors were transfected into MCF-7 or 293T cells. We found that the luciferase activity was significantly reduced when the miR-15b-5p mimic was co-transfected with the wt-HPSE2 reporter plasmid. However, miR-15b-5p had no statistical effect on the mut-HPSE2 3′ UTR (Figures 6D,E). These data indicate that miR-15b-5p regulated HPSE2 expression by directly targeting the 3′ UTR of HPSE2. We thus confirmed that HPSE2 is a direct target of miR-15b-5p.

### HPSE2 Attenuates the Function of miR-15b-5p in BC Cells

**Figure 7:**
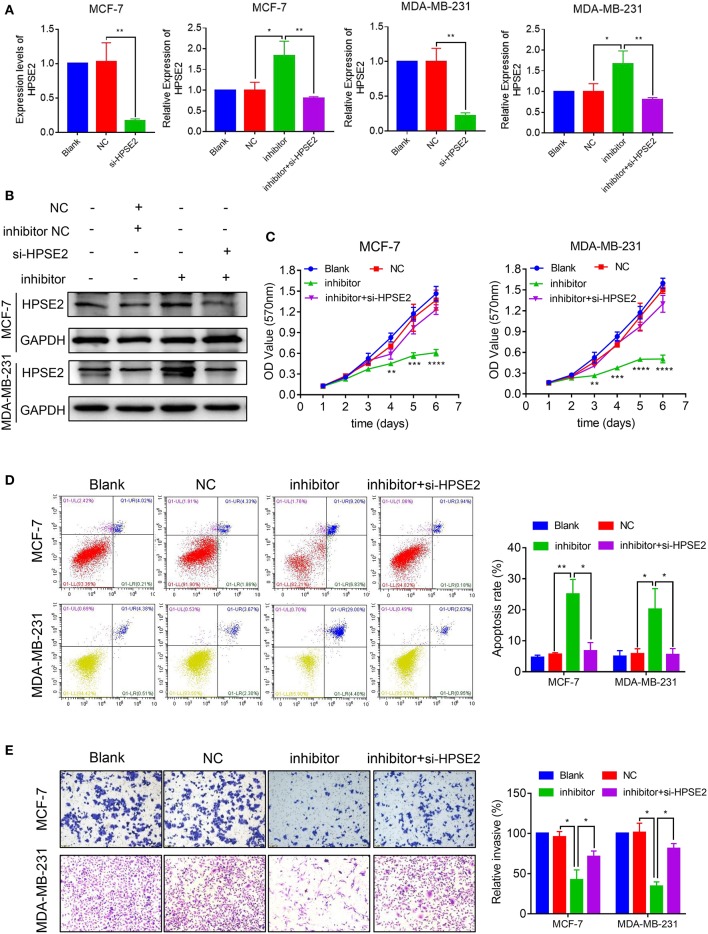
miR-15b-5p/HPSE2 axis may modulate the breast cancer progression. **(A)** The mRNA level of HPSE2 in breast cancer cells underexpressing miR-15b-5p with or without the small interference RNA of HPSE2 (si-HPSE2). **(B)** The protein level of HPSE2 in breast cancer cells underexpressing miR-15b-5p with or without si-HPSE2. **(C)** 3-(4,5-Dimethylthiazol-2-yl)-2,5-diphenyltetrazolium bromide (MTT) assays showed that the effects of miR-15b-5p downregulation on breast cancer cells in inhibiting cell proliferation were significantly reversed by co-transfection of si-HPSE2. **(D)** Flow cytometry showed the apoptotic rates of breast cancer cells transfected with miR-15b-5p inhibitor and/or si-HPSE2. **(E)** The invasion of breast cancer cells underexpressing miR-15b-5p with or without si-HPSE2 (*P < 0.05, **P < 0.01, ***P < 0.001, and ****P < 0.0001).

To determine whether HPSE2 could reverse the oncogenic effects of miR-15b-5p, we co-transfected breast cancer cells with miR-15b-5p inhibitor and HPSE2 siRNA according to the experimental design from previous reported (22–24). The mRNA and protein levels of HPSE2 are shown in Figures 7A,B. When the levels miR-15b-5p and HPSE2 were downregulated simultaneously in either MCF-7 or MDA-MB-231 cells, however, the decreased rates of cell proliferation could be rescued (Figure 7C). The apoptosis rate of breast cancer cells by the miR-15b-5p inhibitor was also reversed after co-transfection with HPSE2 siRNA (Figure 7D). We got similar results from the invasion assay (Figure 7E). These data demonstrate that HPSE2 partially alleviates the oncogenic effects of miR-15b-5p in breast cancer.

### Inhibition of miR-15b-5p Reduces the Tumorigenic Ability of Breast Cancer Cells *in vivo*

**Figure 8:**
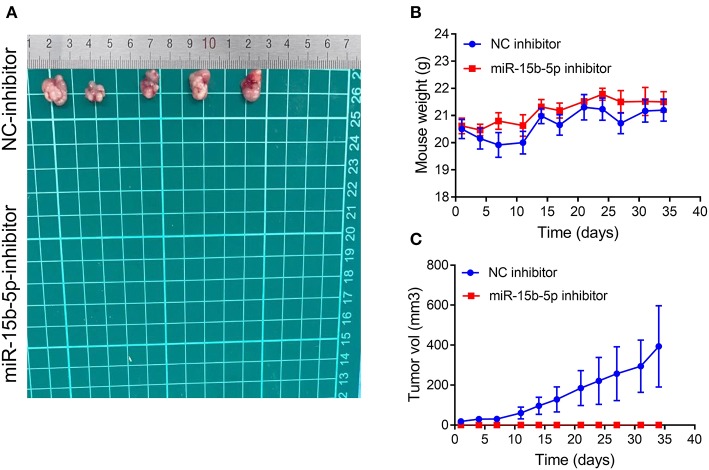
Silencing of miR-15b-5p significantly suppressed tumorigenicity *in vivo*. **(A)** After stable transfection of miR-15b-5p or miR-control in MDA-MB-231 cells, cells are harvested and subcutaneously injected into the right or left flank of female nude mice (*n* = 5); 5 weeks later, nude mice were executed humanely. Tumors are taken for photographing. **(B)** Mice were weighed twice per week. Indicated mouse weights = the mean ± SD. **(C)** Tumor volumes were measured twice per week. The indicated tumor volumes = the mean ± SD.

We tested whether inhibition of miR-15b-5p suppressed tumor formation capacity *in vivo*. The results showed that infection of MDA-MB-231 cells with a lentiviral vector encoding an anti-miR-15b-5p construct resulted in a significant decrease in tumorigenic ability *in vivo* compared to the control group (Figures 8A–C). Therefore, our results indicated that miR-15b-5p acts not only as a simple regulator but also as an essential molecular mechanism for the growth of breast cancer *in vivo*.

## Discussion

In many tumors, miRNAs play an important role in tumor development (25–27). They can negatively regulate the expression of downstream genes and can serve as oncogenes or anti-oncogenes in different tumors (28, 29). Fully one-third of the encoded protein genes is reported to be regulated by miRNAs (30, 31).

Previous studies have reported that miR-15b-5p is upregulated or downregulated in different tumors (11–14, 32, 33). In colorectal cancer and osteosarcoma, miR-15b-5p was downregulated compared with their adjacent normal tissues, and it suppressed the progression and metastasis of cancer (12, 29, 32). On the other hand, miR-15b-5p was found to be upregulated in gastric cancer and prostate cancer tissues (11, 13). These studies demonstrated that miR-15b-5p levels and functions may be tumor type dependent. With respect to breast cancer, there has been no definite research on the role and function of miR-15b-5p.

Here, we found that miR-15b-5p was significantly overexpressed in human breast cancer samples compared with the adjacent normal tissue via the microarray analysis. Additionally, online databases from the Kaplan–Meier Plotter revealed a close positive correlation between more highly expressed miR-15b-5p and worse OS. These findings prompted us to speculate that miR-15b-5p might function as an oncogene in breast cancer when overexpressed. In order to further study its effect on the proliferation, apoptosis, migration, and invasion of breast cancer cells, overexpression and downregulation of miR-15b-5p levels were investigated in two breast cancer cell lines. The evidence suggested that inhibition of miR-15b-5p suppresses proliferation, migration, and invasion of breast cancer cell lines while promoting apoptosis. The opposite results were obtained after overexpression of miR-15b-5p in breast cancer cell lines. Furthermore, we used lentivirus transfection technology to stably screen inhibitory strains for xenogeny tumorigenesis in nude mice. Inhibition of miRNA-15b-5p in MDA-MB-231 cells repressed tumor formation, which further supports our hypothesis.

Next, we attempted to illuminate the functional mechanisms of miR-15b-5p in breast cancer cells via studying its downstream target. Based on the microarray analysis and bioinformatics analysis, we focused on HPSE2, which has been found to act as a suppressor gene in a variety of tumors including breast cancer (34). The explanation for this can probably be found from the relationship between HPSE2 and heparanase, to which it is highly homologous (35, 36). Heparanase is an extracellular matrix-disrupting enzyme that degrades chains of heparan sulfate and heparan sulfate proteoglycans (37, 38). Cancer cells can release a large amount of heparanase, which degrades the basement membrane of blood vessels and the extracellular matrix, thus promoting invasion and metastasis of tumor cells (37). It also induces angiogenesis by activating growth factors, inhibits activated T lymphocytes (37), and modifies the tumor microenvironment (39).

All of these tumor-promoting effects of heparanase are opposed by HPSE2, which lacks the ability to degrade heparan sulfate and importantly has the ability to inhibit heparanase by competing with heparanase for heparan sulfate (40–42).

HPSE2 also promotes tumor suppression by downregulating the transcription factor Id1, further inhibiting heparanase activity and stimulating endoplasmic reticulum stress (43). Downregulation of the transcription factor Id1 reduces vascular endothelial growth factor A (VEGF-A) and lymphatic endothelial growth factor C (LEGF-C), thereby inhibiting tumor angiogenesis and growth, and it facilitates cell differentiation through upregulating the expression of cytokeratin 13 or 15 (44–46). Through all these mechanisms, HPSE2 inhibits heparanase and thus lessens its tumor-promoting actions (34, 43, 47). When HPSE2 levels are elevated in response to hypoxia and endoplasmic reticulum stress, which often occur within tumors, additional tumor suppression arises (43).

According to the data from TCGA database, HPSE2 is underexpressed in breast cancer tissues. The same results were obtained with clinical tissue samples. Elevated expression of HPSE2 is positively correlated with a better prognosis for breast cancer. The expression level of miR-15b-5p in breast cancer is negatively correlated with HPSE2. Our results suggested that knockdown of miR-15b-5p significantly increases HPSE2 mRNA and protein expression, whereas the opposite result was found after overexpression of miR-15b-5p. To verify this hypothesis, we found a binding site complementary to the miR-15b-5p subsequence in the HPSE2 3′ UTR. The luciferase assay demonstrated that miR-15b-5p directly bound to the 3′ UTR of HPSE2. It was further confirmed by rescue experiments. However, the detailed underlying mechanisms still need to be further investigated.

In conclusion, this work presented an upregulated expression of miR-15b-5p in breast cancer, which is positively correlated with the poor prognosis in breast cancer patients. Knockdown of miR-15b-5p resulted in growth inhibition *in vitro* and *in vivo* and lessened the migration capabilities and invasiveness of breast cancer cells. This paper shows that miR-15b-5p acts like an oncogene in breast cancer cell lines by targeting HPSE2 directly. Therefore, the oncogenicity of miR-15b-5p on the tumorigenesis of breast tumor cell lines shown in this work could be credited in part to its modulation of HPSE2. The objective of this research was to explore the possible molecular mechanism of miR-15b-5p in breast carcinoma progression. The results indicate that miR-15b-5p might be a promising diagnostic and prognostic marker of breast cancer and further that it may be a valuable therapeutic target in breast cancer.

## Data Availability Statement

The datasets generated for this study can be found in the NCBI Gene Expression Omnibus (GSE143564).

## Ethics Statement

The studies involving human participants were reviewed and approved by Ethics Committee of Zhongnan Hospital of Wuhan University. The patients/participants provided their written informed consent to participate in this study. This animal study was reviewed and approved by the Center for Animal Experiment of Wuhan University and the ABSL-III Laboratory of Wuhan University.

## Author Contributions

BW, GL, TY, YJ, DZ, and LD performed the *in vitro* assays. BW, YP, and TY did the *in vivo* studies. GL and YP collected clinical samples. BW, GL, and YP analyzed the data and wrote the manuscript. YP and YW designed this study. YP, FZ, and YW reviewed the manuscript. All authors read and approved the final manuscript.

### Conflict of Interest

The authors declare that the research was conducted in the absence of any commercial or financial relationships that could be construed as a potential conflict of interest.
