# Supplementary material for: miR-15b-5p Promotes Growth and Metastasis in Breast Cancer by Targeting HPSE2
Source: Front Oncol. 2020 Feb 26;10:108. doi: 10.3389/fonc.2020.00108 (PMC7054484; doi:10.3389/fonc.2020.00108)
Supplement: Supplementary file 1 [file Data_Sheet_1.pdf]

Supplementary Material

Table S1 - Clinical information of human samples used in microarray.

Samples	Gender	Age	pathology type	Molecular type	T stage
1	Female	59	invasive ductal carcinoma	HER2+/ER+	2
2	Female	42	invasive ductal carcinoma	Luminal	3
3	Female	60	invasive ductal carcinoma	Luminal	3

HER2+, human epidermal growth factor receptor-2; ER, estrogen receptor.

Table S2 - Clinical information of human samples used for western blot.

Samples	Gender	Age	Pathology type	Molecular type	T stage
1	Female	45	invasive ductal carcinoma	HER2+	1
2	Female	32	invasive ductal carcinoma	Luminal	1
3	Female	75	invasive ductal carcinoma	Luminal	1
4	Female	57	invasive ductal carcinoma	Luminal	2
5	Female	40	invasive ductal carcinoma	HER2+	2
6	Female	82	invasive ductal carcinoma	HER2+	1

Table S3 - Sequences of primers and microRNAs.

Primer/microRNA		Sequence
miR-15b-5p	F	5'-UAGCAGCACAUCAUGGUUUACA-3'
	R	5'-CTCAACTGGTGTCGTGGA-3'
U6	F	5'-CTCGCTTCGGCAGCACAT-3'
	R	5'-ACGCTTCACGAATTTGCGT-3'
NC	F	5’-UUCUCCGAACGUGUCACGUTT-3’
	R	5’-ACGUGACACGUUCGGAGAATT-3’
miR-15b-5p mimics	F	5’-UAGCAGCACAUCAUGGUUUACA-3’
	R	5’-UAAACCAUGAUGUGCUGCUAUU-3’
miR-15b-5p inhibitor		5’-UGUAAACCAUGAUGUGCUGCUA-3’
NC inhibitor		5’-CAGUACUUUUGUGUAGUACAA-3’
HPSE2 siRNA	sense	5’-GCUGGAUCCGUCCAUCAUUTT-3’
	antisense	5’-AAUGAUGGACGGAUCCAGCTT-3’
HPSE2	F	5’-GCTCTGTCTACAGGCAAGGG-3’
	R	5’-GGGAGTAAGT TAGGGAGACT-3’
GAPDH	F	5’-AGGTGAAGGTCGGAGTCAACG-3’
	R	5’-GCTCCTGGAAGATGGTGATGG-3’

miR, microRNA; NC, negative control; F, forward; R, reverse.

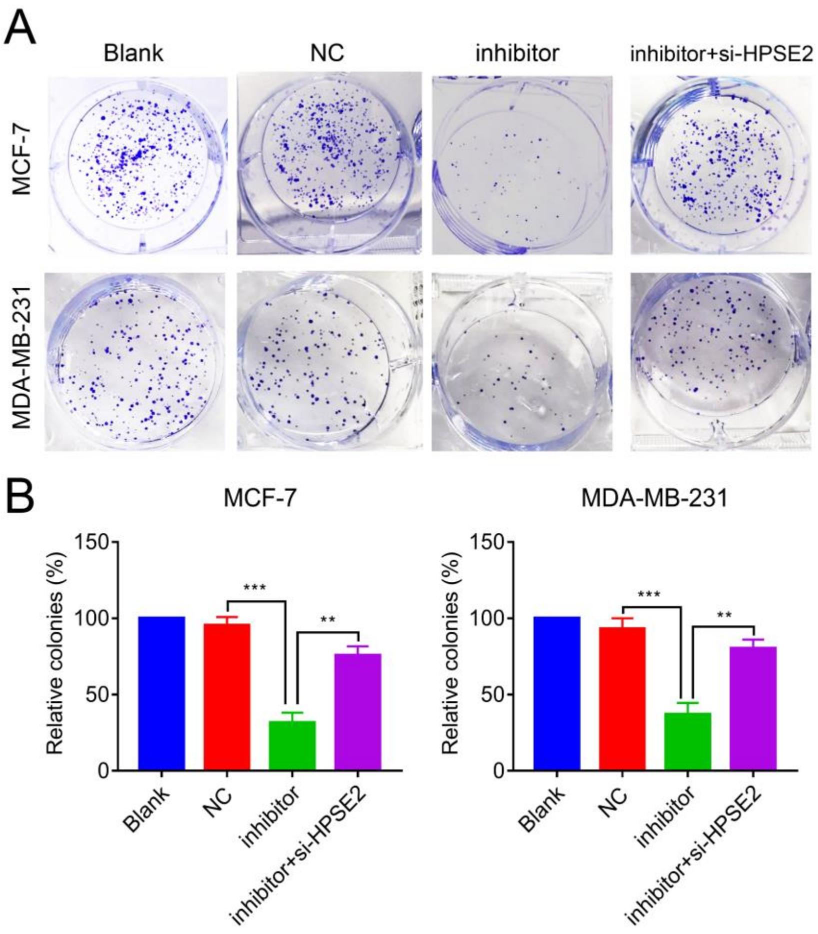

Figure S1. **miR-15b-5p/HPSE2 axis modulates the breast cancer Progression**.(A,B) Colony formation assays demonstrated that the effects of miR-15b-5p downregulation on breast cancer cells in inhibiting cells' proliferation were significantly reversed by co-transfection of si-HPSE2.
